# Beyond the Norm: Gastroenteritis-Induced Atypical Hemolytic Uremic Syndrome in the Absence of Complement Dysfunction

**DOI:** 10.7759/cureus.72182

**Published:** 2024-10-23

**Authors:** Rita Bernardino, Rodrigo Leão, Angela Ghiletchi, Carolina Coelho, Beatriz Sampaio

**Affiliations:** 1 Internal Medicine, Hospital Curry Cabral - Centro Hospitalar Universitário de Lisboa Central, Lisboa, PRT; 2 Internal Medicine, NOVA Medical School, Lisboa, PRT; 3 Internal Medicine, Unidade Local de Saúde de São José, Lisboa, PRT; 4 Internal Medicine, Centro Hospitalar Universitário de Lisboa Central, Lisboa, PRT

**Keywords:** acute kidney injury (aki), atypical hemolytic-uremic syndrome (ahus), gastroenteritis, normal complement system, thrombotic microangiopathy (tma)

## Abstract

Atypical hemolytic uremic syndrome (aHUS) is a complex disorder characterized by thrombotic microangiopathy, typically driven by complement dysregulation. While most cases of aHUS are linked to genetic abnormalities in the complement system, sporadic instances occur without identifiable genetic or complement involvement. This report discusses the case of a 38-year-old woman who developed aHUS following gastroenteritis, marked by acute kidney injury requiring dialysis, yet displayed normal complement levels. This case highlights the diagnostic and management challenges in aHUS when traditional disease markers are absent and emphasizes the importance of tailored therapeutic approaches.

## Introduction

Atypical hemolytic uremic syndrome (aHUS) is a complex disorder primarily driven by dysregulation of the complement system, leading to thrombotic microangiopathy (TMA). While most research and clinical approaches focus on the complement-mediated pathways, aHUS without clear complement involvement represents a diagnostic and therapeutic challenge [1]. This subset of patients may not require standard treatments, such as plasma therapy or complement inhibitors like eculizumab, with management shifting toward eliminating the underlying trigger whenever possible [2-4].

The current case report details the unusual presentation of a 38-year-old woman who developed aHUS following an episode of gastroenteritis. Unlike the more common presentations involving abnormal complement activation, this patient exhibited normal complement levels, adding complexity to the diagnosis and management. The absence of complement dysregulation necessitated a departure from the typical use of complement inhibitors, directing focus toward supportive care and renal management. This case contributes to the literature by documenting an instance of aHUS triggered by an alternative pathophysiological mechanism, potentially linked to an infectious precipitant rather than genetic or inherent complement abnormalities [3].

## Case presentation

A 38-year-old woman with no significant medical history presented to the emergency department with a one-week history of severe abdominal pain, nausea, vomiting, and diarrhea. Initially, her symptoms were consistent with gastroenteritis, but she soon developed progressive fatigue, shortness of breath, and dark-colored urine. Her examination on admission revealed pallor, mild jaundice, and lower extremity edema, with a blood pressure of 101/58 mmHg, a pulse rate of 105 beats per minute, and a respiratory rate of 18 breaths per minute. Laboratory tests were urgently performed given the constellation of symptoms suggesting systemic illness.

Initial laboratory workup demonstrated elevated C-reactive protein (CRP), indicating a significant inflammatory process. Additionally, the patient had thrombocytopenia, as evidenced by a significantly reduced platelet count, and mild elevations in liver enzymes (AST and ALT). Her bilirubin was mildly elevated, specifically the indirect fraction, suggesting hemolysis as a contributing factor. The most concerning finding was acute kidney failure, characterized by a marked rise in serum creatinine and blood urea nitrogen (BUN), consistent with significant renal impairment (Table 1). Despite hydration and supportive care, the patient became oliguric, necessitating the initiation of hemodialysis.

**Table 1:** Progression of laboratory values LDH: lactate dehydrogenase; CRP: C-reactive protein; AST: aspartate aminotransferase; ALT: alanine aminotransferase Reference ranges: Hemoglobin: 12-16 g/dL, Platelet count: 150,000-450,000/μL, Serum creatinine: 0.6-1.1 mg/dL, LDH: 135-214 U/L, CRP: <5 mg/L, Liver enzymes (AST): 8-33 U/L, (ALT): 7-56 U/L

Timepoint	Hemoglobin (g/dL) reference value	Platelet count (/μL)	Serum creatinine (mg/dL)	LDH (U/L)	CRP (mg/L)	AST/ALT (U/L)
Admission	9	55	4	700	100	48/64
Day 2	8.6	50	5.3	800	110	44/62
Day 4	7.5	45	7.9	820	90	40/55
Week 1	8.5	48	4.3	760	85	35/50
Week 2	9.5	75	3.8	620	60	30/45
Week 4 (discharge)	10	120	2	400	30	28/40
3-month follow-up	12	150	0.8	190	5	25/35

Further laboratory analysis confirmed the presence of microangiopathic hemolytic anemia. A peripheral blood smear revealed numerous schistocytes, fragmented red blood cells characteristic of TMA. Serum lactate dehydrogenase (LDH) was markedly elevated, further supporting the diagnosis of hemolysis (Table 1), while haptoglobin levels were undetectably low, reinforcing the diagnosis of hemolytic anemia (Table 2). Given the rapid progression of anemia (Table 1). The patient required a transfusion of one unit of packed red blood cells.

**Table 2:** Results of further diagnostic tests

Test	Result
C3 complement	Normal
C4 complement	Normal
CH50	Normal
AH50	Normal
Factor B	Normal
Factor I	Normal
Factor H	Normal
ADAMTS13 activity	Normal
Cultures for Salmonella, Shigella, E. coli O157	Negative
HIV serology	Negative
Hepatitis serology	Negative
Autoimmune panel	Negative
Coombs test	Negative
Haptoglobin	Very low

Additionally, serological tests for recent streptococcal infection, including antistreptolysin O (ASO) titer and anti-DNase B antibody, were negative, excluding post-infectious glomerulonephritis as a potential cause of her renal dysfunction. Amylase levels were also normal, ruling out pancreatitis as a contributor to her acute illness. Imaging studies, including abdominal computed tomography (CT) and renal ultrasound, showed no structural abnormalities in the kidneys or other organs.

A renal biopsy was performed to further investigate the cause of the acute kidney injury (AKI). Histopathological examination showed classic features of TMA, including endothelial cell swelling and subendothelial widening of the glomerular capillary walls. There was also evidence of fibrin thrombi in the small vessels of the kidney, but no immune complex deposition, confirming the absence of an immune-mediated process. These findings were consistent with a diagnosis of aHUS.

Despite the typical features of aHUS, complement studies were thoroughly evaluated and found to be normal. Specifically, levels of complement components C3 and C4 were within reference ranges, as were functional complement assays including CH50 and AH50. Factor H, Factor I, and Factor B, key regulators of the complement system, were also found to be normal, further excluding complement-mediated pathways (Table 2). Given these results, complement-inhibiting therapy with eculizumab was not pursued, and plasma exchange therapy was deemed unnecessary.

Extensive infectious and autoimmune workup was performed to exclude secondary causes of TMA. Stool cultures for gastrointestinal pathogens, including *Salmonella*, *Shigella*, and *Escherichia coli* O157 were negative, ruling out typical hemolytic uremic syndrome (HUS) associated with Shiga toxin-producing *E. coli*. ADAMTS13 activity was measured and found to be within normal limits, ruling out thrombotic thrombocytopenic purpura (TTP). Autoimmune diseases were excluded through a negative workup for antinuclear antibodies (ANA) and anti-double-stranded DNA antibodies, and serologic tests for HIV and hepatitis B and C were negative. Coombs test for direct antiglobulin was negative, excluding immune-mediated hemolytic anemia (Table 2). Given the patient’s age, a pregnancy test was also performed and was negative.

The combination of normal complement activity, negative infectious and autoimmune workups, and characteristic findings on renal biopsy led to a diagnosis of aHUS with no apparent underlying secondary causes. The patient was managed conservatively with supportive care, including intravenous fluids, blood pressure control, and dialysis. Over the course of several weeks, her kidney function gradually improved, and dialysis was eventually discontinued. At follow-up, her hematological parameters had normalized, and she remained in stable condition with no recurrence of hemolysis or thrombocytopenia.

## Discussion

This case of a 38-year-old woman diagnosed with aHUS following gastroenteritis provides important insights into the variability of aHUS presentations. It highlights key considerations in its diagnosis and management. aHUS is typically a rare, life-threatening disorder characterized by TMA involving hemolysis, thrombocytopenia, and AKI. The pathophysiology of aHUS generally involves dysregulation of the alternative complement pathway, but this case is remarkable because the patient exhibited normal complement function, which represents an atypical mechanism of aHUS.

In most cases, aHUS is caused by genetic mutations leading to uncontrolled complement activation, which results in endothelial damage, platelet aggregation, and formation of microthrombi, particularly affecting the kidneys. However, the absence of complement dysregulation in this case raises important questions about the mechanisms of endothelial injury and TMA. This patient presented with classic signs of aHUS, including anemia, thrombocytopenia, and AKI, but had normal complement levels (C3, C4, CH50, AH50) and normal complement regulatory proteins (Factor H, Factor I, and Factor B). This suggests that in some instances, aHUS may be triggered by factors other than complement dysregulation, possibly related to the preceding gastroenteritis [4].

The role of gastroenteritis as a triggering event in this patient is significant, as it is a well-recognized precursor of typical HUS caused by Shiga toxin-producing E. coli (STEC) [1]. However, stool cultures for E. coli O157, Salmonella, and Shigella were negative, ruling out typical HUS. This emphasizes the importance of differentiating between typical HUS, which is infection-related, and atypical HUS, which is more complex and less predictable. Gastroenteritis may have acted as a nonspecific trigger in this patient, leading to endothelial injury and TMA without complement involvement [4].

The diagnosis of aHUS in the absence of complement dysregulation presents a significant challenge [5]. The clinical presentation of TMA was clear, with the presence of schistocytes on the peripheral smear, elevated LDH, and low haptoglobin indicating hemolysis, along with AKI and thrombocytopenia. However, the extensive workup, including normal ADAMTS13 levels, ruled out TTP and normal complement activity excluded the typical complement-mediated aHUS pathway. Additionally, the negative workup for autoimmune diseases, HIV, and hepatitis ruled out secondary causes of TMA, while normal Coombs tests confirmed that the hemolysis was not immune-mediated. This thorough exclusion process reinforced the diagnosis of aHUS despite the lack of complement abnormalities.

The renal biopsy findings of TMA, including endothelial swelling and subendothelial widening, confirmed the diagnosis of TMA and provided histological evidence supporting the diagnosis of aHUS. The absence of immune complex deposition further ruled out immune-mediated kidney injury, such as lupus nephritis or vasculitis. The renal involvement in this case was severe, leading to the need for dialysis, but the gradual improvement in renal function over time highlights the potential for recovery even in severe cases of aHUS.

The management of aHUS typically involves the use of eculizumab, a monoclonal antibody that inhibits complement component C5, halting the progression of complement-mediated endothelial damage [2,5]. However, in this case, eculizumab was not used, not only because the patient’s complement system was normal but also due to the lack of evidence supporting its effectiveness in such scenarios, as well as the potential risks associated with its use, particularly the increased susceptibility to infections like meningococcal disease [5]. While eculizumab has significantly improved outcomes for selected patients with aHUS, its benefits must be carefully weighed against the risks in patients where complement activation is not clearly implicated [5]. Plasma therapy was also not initiated because it was unlikely to benefit this patient due to the normal complement activity and the absence of an identified antibody-mediated mechanism, along with the risks that such therapy could pose. This highlights the importance of a personalized approach to treating aHUS. The patient was managed with supportive care, including dialysis and a single blood transfusion for anemia, eventually achieving recovery of renal function without the need for eculizumab or plasma exchange.

## Conclusions

This case of aHUS following gastroenteritis highlights the complexity of diagnosing and managing thrombotic microangiopathies, particularly in patients with normal complement function. It emphasizes the need for a thorough diagnostic workup to rule out secondary causes of TMA and underscores the importance of tailoring treatment to the individual patient. In cases where complement dysregulation is absent, conservative management with supportive care may lead to favorable outcomes. This case contributes to the growing understanding of aHUS as a heterogeneous disease and suggests that complement-independent mechanisms may play a role in some cases, necessitating ongoing research into alternative therapeutic strategies.
